# A Novel *De Novo EFNB1* Gene Mutation in a Mexican Patient with Craniofrontonasal Syndrome

**DOI:** 10.1155/2013/349725

**Published:** 2013-02-21

**Authors:** M. A. Ramirez-Garcia, O. F. Chacon-Camacho, C. Leyva-Hernandez, A. Cardenas-Conejo, J. C. Zenteno

**Affiliations:** ^1^Genetics Department, UMAE Hospital de Pediatría, Centro Médico Nacional Siglo XXI, IMSS, Cuauhtémoc 330, Colonia Doctores, 06720 México, DF, Mexico; ^2^Genetics Department and Research Unit, Instituto de Oftalmología “Conde de Valenciana,” Chimalpopoca 14, Colonia Obrera, 06800 México, DF, Mexico; ^3^Genetics Department, Hospital General Gaudencio González Garza, Centro Médico Nacional La Raza, IMSS, Calzada Vallejo, Colonia Azcapotzalco, 02990 México, DF, Mexico; ^4^Department of Biochemistry, Faculty of Medicine, UNAM, México, DF, Mexico

## Abstract

Craniofrontonasal syndrome (CNFS) is an X-linked disorder caused by mutations in the *EFNB1* gene in which, paradoxically, heterozygous females are more severely affected than hemizygous males. In this paper, the clinical and molecular studies of a female subject with CFNS are described. A novel *de novo* c.473T>C (p.M158T) mutation in exon 3 of *EFNB1* was demonstrated in this patient. The M158 residue of the Ephrin-B1 protein is highly conserved between species. Our results expand the mutational spectrum exposed by CNFS.

## 1. Introduction

Craniofrontonasal syndrome (CFNS; *OMIM # 304110*) [1] is an X-linked syndrome involving developmental malformation with variable clinical expression characterized by severe hypertelorism, depressed nasal bridge and bifid nasal tip, frontal bossing, coronal suture synostosis, corpus callosum agenesis, and occasionally cleft lip or palate [2–4]. This disorder is caused by mutations in the *EFNB1* gene, located at Xq13.1, and encoding a ligand of the Ephrin family of receptor protein tyrosine kinases [3]. The most common types of *EFNB1 *mutations (up to 55%) in CFNS patients are frameshift, nonsense, and splice site mutations that lead to premature termination codons (PTCs). Missense mutations constitute approximately 42% of all *EFNB1 *mutations, and most of them occur in exons 2 and 3, leading to the substitutions of amino acid residues involved in receptor-ligand interaction and cell signaling, which are critical for cell sorting, migration and adhesion, midline fusion, axon guidance, neural plasticity, and synaptogenesis [5–8]. Here, we describe a sporadic case of CFNS due to a novel *EFNB1* mutation occurring in a female Mexican patient.

## 2. Case Presentation

**Figure 1:**
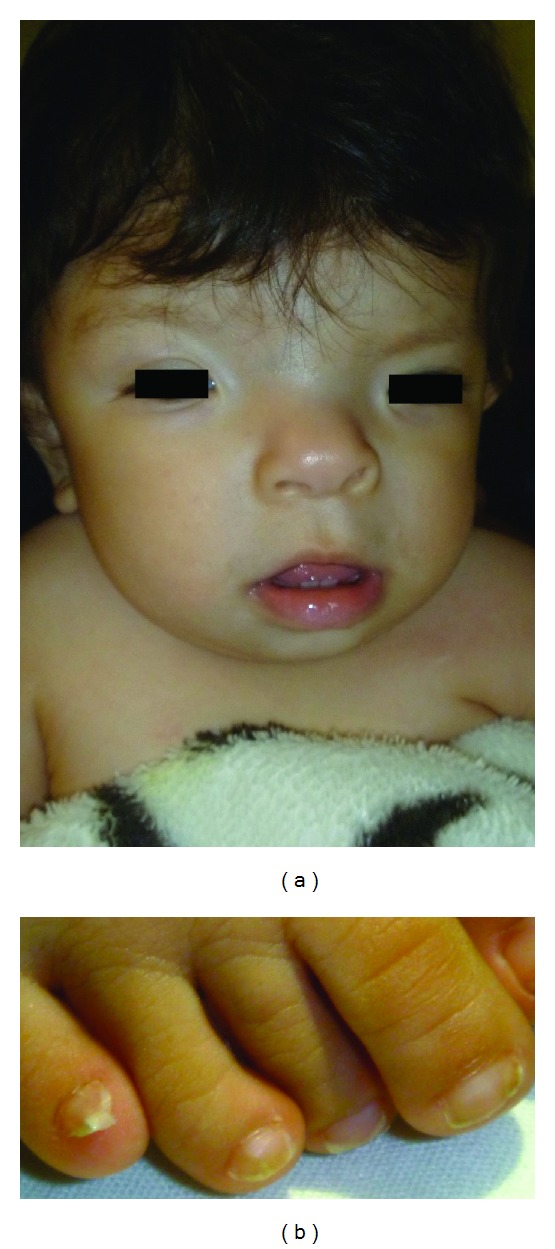
(a) Patient's facial appearance. Hypertelorism, broad and flattened nasal bridge, and bifid nasal tip are evident. (b) Broad toes with longitudinally split nails can be observed.

A 3-month-old girl was referred to the Genetics Department after a frontoorbital advancement surgery due to right unicoronal synostosis and facial dysmorphism. She is the only child of healthy, nonconsanguineous parents. There was no prenatal exposure to teratogenic agents. A structural ultrasound at 24 weeks revealed a nonspecific cranial malformation. The patient was delivered by caesarean section at 38 weeks of pregnancy and had a birth weight of 2,700 g, birth length of 48.5 cm, and an Apgar score of 8/9. Birth examination disclosed plagiocephaly (left frontal bossing and right coronal synostosis), hypertelorism, downslanting palpebral fissures, facial asymmetry, a broad and flattened nasal bridge, a bifid nasal tip, and broad thumbs and halluces with longitudinally split nails (Figure 1). No abnormalities were found in the transfontanellar ultrasound and echocardiogram performed during the first week of life. Cytogenetic study was normal (46,XX[25]). When the patient reached the age of 3 months, cranial computerized tomography (CT) revealed right coronal synostosis and mild compression of the surrounding cerebral parenchyma, which prompted surgery. At present, she has reached adequate development milestones and growth parameters.

After obtaining local ethics institutional approval and the informed consent of her parents, genomic DNA was extracted from the patient's peripheral blood leukocytes using a semiautomated Quickgene system (Fujifilm, Tokyo, Japan). The complete *EFNB1* coding sequence, including the exon-intron boundaries, was amplified by PCR using primers for the 5 exons (Table 1), and direct automated sequencing was performed using the Big Dye Terminator Cycle Sequencing kit (Applied Biosystems, Foster City, California, USA) in an ABI Prism 3130 Genetic Analyzer (Applied Biosystems).

**Figure 2:**
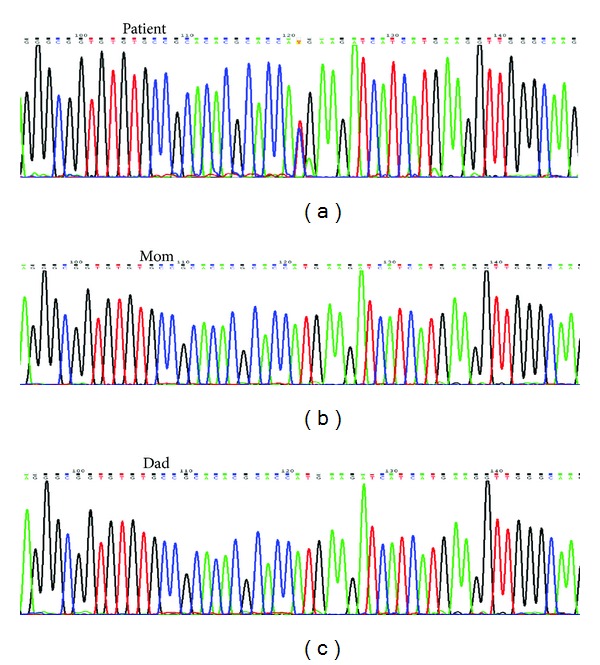
Partial nucleotide sequence of the *EFNB1* gene in DNA from patient (a), proband's mother (b), and proband's father (c). (a) A heterozygous T to C transition at nucleotide position c.473 in exon 3 predicting a methionine (ATG) to threonine (ACG) replacement at residue 158 (p.M158T) is shown. (b and c) Normal nucleotide (T) in both parents.

Nucleotide analysis disclosed a novel heterozygous transition c.473T>C in exon 3 of *EFNB1*. This mutation predicted a substitution of methionine (ATG) to threonine (ACG) in the extracellular domain of the protein (p.M158T) (Figure 2(a)). Both parents had a normal sequence (Figures 2(b) and 2(c)).

## 3. Discussion

Our report discussed a patient with clinical characteristics consistent with CNFS and in whom a novel *de novo EFNB1* mutation was demonstrated. CFNS shows a phenotypic pattern not usually seen in X-linked disorders, as heterozygous females are more severely affected than hemizygous males. Mutations in *EFNB1 *are the cause of CFNS in the majority of patients, with a mutation detection rate of 92% [9, 10]. CNFS's clinical manifestations are sex dependent, with multiple skeletal malformations in affected females and mild or no malformations in male carriers. Recently, the severe phenotype in females has been explained through the cellular interference hypothesis; cellular interference is caused by the combination of Ephrin ligand/receptor promiscuity and the consequences of random X inactivation in distinct cellular compartments [3, 11]. Although we have identified a novel *de novo* mutation, no other new clinical features were found in the physical examination.

The* EFNB1 *gene encodes Ephrin-B1 protein, a member of the ephrin family of transmembrane ligands for Eph receptors with tyrosine kinase activity. These proteins play a crucial role in cell migration and pattern formation during embryonic development [12]. Missense mutations, such as that demonstrated in our patient, constitute about 42% of *EFNB1* mutations. There are non-*hotspot* mutations in *EFNB1 *gene; however, most of reported substitutions occur in the extracellular domain, which is encoded by exons 2 and 3, leading to a change in amino acid residues, which are important for receptor-ligand interaction and signaling, and cause loss of function [7]. 

**Figure 3:**
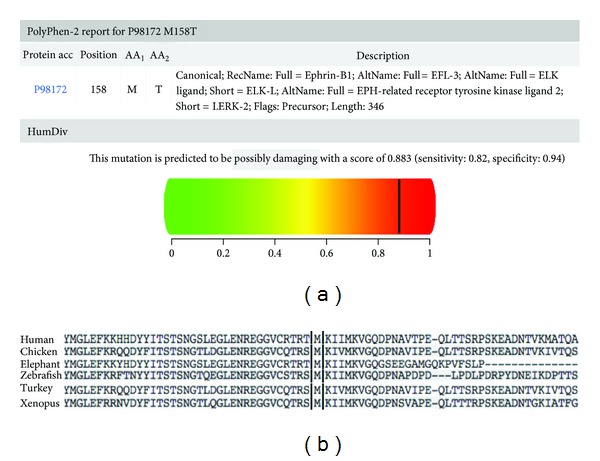
*In silico* analysis using *PolyPhen-2* software shows that change in p.M158T is pathogenic (a) and predicted to be highly conserved in different species (b).

The replacement from a hydrophobic sulfur amino acid such as methionine for a hydrophilic amino acid, threonine, modifies the polarity of the protein. *In silico* analysis of this novel missense *EFNB1* mutation using *PolyPhen-2* software indicated that this change is pathogenic (Figure 3(a)) and predicted to be highly conserved in different species (Figure 3(b)) [13]. The fact that this nucleotide substitution-transition was not found in the NHLBI Exome Sequencing Project (ESP; Exome Variant Server) supports the novel condition of our mutation [14].

In conclusion, we presented a patient with craniofrontonasal syndrome due to a novel *de novo* heterozygous transition mutation c.473T>C in exon 3 of *EFNB1*. Our results expand the *EFNB1* mutational spectrum in CNFS patients. The M158 residue (methionine) of the Ephrin-B1 protein is highly conserved between species.

## Figures and Tables

**Table 1:** PCR and sequencing primers for *EFNB1*.

Primer name	5′-3′ orientation	Length of PCR product (bp)
EFNB1-ex1-F	AAGGCGAGGCGAGCTTTGGT	318
EFNB1-ex1-R	AAGCCGGAGACAAAATGAGG	
EFNB1-ex2-F	TTGTCCGCTTCCCTGGTTCT	445
EFNB1-ex2-R	ATTGCACCACTTAGAAGCTCC	
EFNB1-ex3-F	GCTGAAGCAGAATGGGAGTT	246
EFNB1-ex3-R	GCCAGGAACATCTGTTCCAA	
EFNB1-ex4-F	GGTTACAGTATCCAGGCCAT	312
EFNB1-ex4-R	GCCCAGCTTGCATTTCTTCA	
EFNB1-ex5-F	TGAAGAAATGCAAGCTGGGC	606
EFNB1-ex5-R	ATACAAAGGTGGGCACAGCT	

## References

[1] OMIM (TM) Bethesda MD McKusick-Nathans Institute for Genetic Medicine, John Hopkins University, National Center for Biotechnology Information, National Library of Medicine.

[2] Wieland I, Jakubiczka S, Muschke P (2004). Mutations of the ephrin-B1 gene cause craniofrontonasal syndrome. *American Journal of Human Genetics*.

[3] Wieacker P, Wieland I (2005). Clinical and genetic aspects of craniofrontonasal syndrome: towards resolving a genetic paradox. *Molecular Genetics and Metabolism*.

[4] Saavedra D, Richieri-Costa A, Guion-Almeida ML (1996). Craniofrontonasal syndrome: study of 41 patients. *American Journal of Medical Genetics A*.

[5] Twigg SRF, Matsumoto K, Kidd AMJ (2006). The origin of EFNB1 mutations in craniofrontonasal syndrome: frequent somatic mosaicism and explanation of the paucity of carrier males. *American Journal of Human Genetics*.

[6] Twigg SRF, Kan R, Babbs C (2004). Mutations of ephrin-B1 (EFNB1), a marker of tissue boundary formation, cause craniofrontonasal syndrome. *Proceedings of the National Academy of Sciences of the United States of America*.

[7] Makarov R, Steiner B, Gucev Z, Tasic V, Wieacker P, Wieland I (2010). The impact of CFNS-causing EFNB1 mutations on ephrin-B1 function. *BMC Medical Genetics*.

[8] Wieland I, Makarov R, Reardon W (2008). Dissecting the molecular mechanisms in craniofrontonasal syndrome: differential mRNA expression of mutant EFNB1 and the cellular mosaic. *European Journal of Human Genetics*.

[9] Wallis D, Lacbawan F, Jain M (2008). Additional EFNB1 mutations in craniofrontonasal syndrome. *American Journal of Medical Genetics A*.

[10] Wieland I, Reardon W, Jakubiczka S (2005). Twenty-six novel EFNB1 mutations in familial and sporadic craniofrontonasal syndrome (CFNS). *Human Mutation*.

[11] Zafeiriou DI, Pavlidou EL, Vargìami E (2011). Diverse clinical and genetic aspects of craniofrontonasal syndrome. *Pediatric Neurology*.

[12] Klein R (2004). Eph/ephrin signaling in morphogenesis, neural development and plasticity. *Current Opinion in Cell Biology*.

[13] http://genetics.bwh.harvard.edu/pph2/.

[14] http://evs.gs.washington.edu/EVS/.

